# Molecular characterization and serodiagnostic potential of two serpin proteins in *Psoroptes ovis* var. *cuniculi*

**DOI:** 10.1186/s13071-020-04501-8

**Published:** 2020-12-11

**Authors:** Xiaobin Gu, Yuhang Chen, Chongyang Zhang, Yue Xie, Nengxing Shen, Ce Wang, Xuan Zhou, Guangyou Yang, Ran He, Xuerong Peng, Deying Yang, Zhi He, Zhijun Zhong

**Affiliations:** 1 Department of Parasitology, College of Veterinary Medicine, Sichuan Agricultural University, Chengdu, 611130 Sichuan People’s Republic of China; 2 Mianyang Animal Disease Control Center, Mianyang, 621000 Sichuan People’s Republic of China; 3 Institute of Animal Genetics and Breeding, College of Animal Science and Technology, Sichuan Agricultural University, Ya’an, 625014 Sichuan People’s Republic of China; 4 Department of Chemistry, College of Life and Basic Science, Sichuan Agricultural University, Ya’an, 625014 Sichuan People’s Republic of China

**Keywords:** *Psoroptes ovis* var. *cuniculi*, Serpin, Transcriptional level, Tissue localization, Indirect ELISA, Serodiagnosis

## Abstract

**Background:**

*Psoroptes ovis* var. *cuniculi* is a common ectoparasite of wild and domestic rabbits worldwide that causes economically devastating losses in commercial rabbit husbandry and significantly affects the overall health of rabbits. Serine proteinase inhibitor (serpin) is present in almost all organisms that are involved in host–pathogen interactions, inflammatory responses, and reproductive development, among others. However, very little research has been carried out on *P. ovis* var. *cuniculi* serpins.

**Methods:**

Two serpin genes of *P. ovis* var. *cuniculi* (Pso c 27 and PsoSP2 cDNAs) were cloned and molecularly characterized. The transcriptional profiles and tissue localization of these two serpins in *P. ovis* var. *cuniculi* were investigated by quantitative real-time PCR and immunohistochemistry, respectively. The potential function of recombinant Pso c 27 and PsoSP2 (rPso c 27 and rPsoSP2) in the serodiagnosis of *P. ovis* var. *cuniculi* infestation in rabbits was evaluated using a newly devleoped indirect enzyme-linked immunosorbent assay.

**Results:**

Both the 523-residue Pso c 27 and the 240-residue PsoSP2 proteins contained typical serpin domains and signatures. Both Pso c 27and PsoSP2 cDNAs were expressed throughout the life-cycle; specifically, the cDNAs showed significantly higher expression in female mites than in larva, nymph, and male mites (Pso c 27: *F*_(3, 8)_ = 1935.953, *P* < 0.0001; PsoSP2: *F*_(3, 8)_ = 660.669, *P* < 0.0001). The native Pso c 27 and PsoSP2 proteins localized in the ovary and mouthparts of adult female mites, respectively. Compared to rPsoSP2, rPso c 27 showed better diagnostic efficiency, with higher values of sensitivity, specificity, and area under the receiver operating characteristic curve (rPso c 27 *vs* rPsoSP2: 96.0 *vs* 90.0%; 90.91 *vs* 78.18%; 0.988 *vs* 0.964, respectively). Moreover, rPso c 27 showed seropositivity in 80% of the rabbits as early as the 2 weeks post-infestation, prior to visible clinical signs and microscopy-positive of skin scrapings.

**Conclusions:**

These results suggest that these two serpins may play essential roles in reproductive development, serum-feeding, and pathogenicity of *P. ovis* var. *cuniculi*. Compared to PsoSP2, Pso c 27 appears to be a potential antigen for serodiagnosis of *P. ovis* var. *cuniculi* infestation in rabbits, especially at the early stage of infestation. 
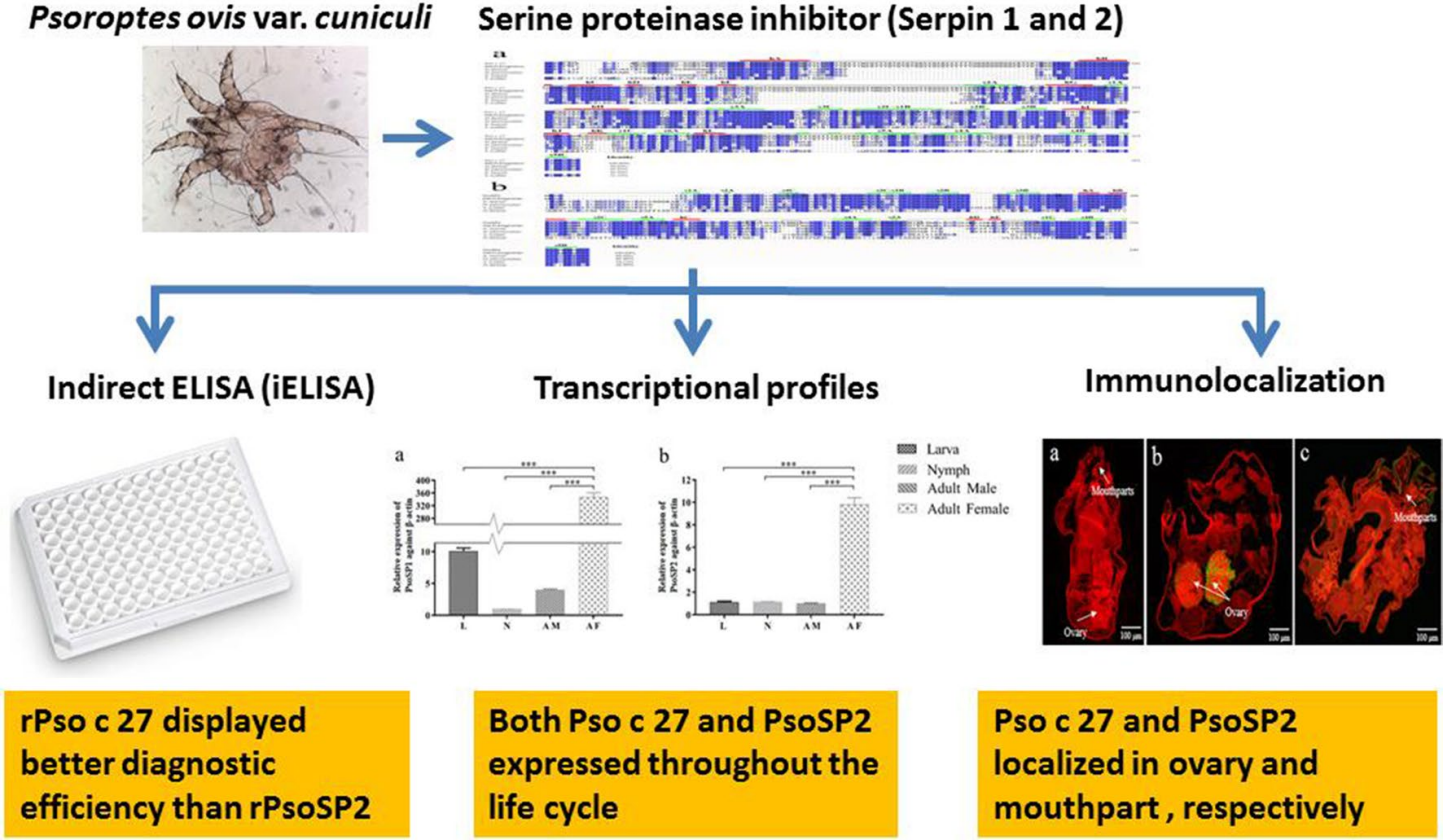

## Background

*Psoroptes ovis* var. *cuniculi* is a common ectoparasite of wild and domestic rabbits worldwide [1, 2]. This mite causes psoroptic mange in rabbits, which mainly presents as intense cutaneous inflammation, extreme pruritus, and crusted skin lesions [1, 2]. It signficantly affects the health welfare of rabbits and causes severe economic losses in commercial rabbit husbandry [2, 3].

The obligate ectoparasite *P. ovis* var. *cuniculi* is a non-burrowing mite that spends its entire life on the surface of host skin [4] where it feeds on serous fluids, lymph, and red blood cells [5]. The mite produces essential proteins to resist the host complement system to ensure successful feeding and self-proliferation. It also excretes allergens to promote the subsequent cutaneous inflammatory response [6, 7]. Serine protease inhibitor (serpin) is expressed in almost all organisms. In arthropods, it has shown to possess a variety of fundamental physiological functions including anticoagulation, regulation of the inflammation response, and reproductive development, among others [8]. It also plays an essential role in the host–pathogen interaction [9]. Additionally, serpin may serve as a promising diagnostic antigen or vaccine candidate [10, 11].

Recently, our analysis of transcriptomic data revealed that serpins exist in *P. ovis* var. *cuniculi* [12], but to our knowledge no further research on *P. ovis* var. *cuniculi* serpins has been reported to date. Analysis of these transcriptomic data led to the identication of two serpin genes of *P. ovis* var. *cuniculi* (Pso c 27 and PsoSP2 cDNAs) [12]. In the present study we focused on the function of these genes. We cloned and expressed the two recombinant Pso c 27 and PsoSP2 in prokaryotic expression vectors and performed sequence analysis. We also investigated the transcriptional profiles as well as tissue localization in mites, and their potential efficiencies in the diagnosis of *P. ovis* var. *cuniculi* infestation in rabbits were determined by indirect enzyme-linked immunosorbent assay (iELISA). This is a preliminary study aimed at elucidating the roles of these two proteins in *P. ovis* var. *cuniculi* and increasing our understanding of their functions.

## Methods

### Mite collection and RNA extraction

*Psoroptes ovis* var. *cuniculi* were harvested from an infested New Zealand White rabbit maintained at the Department of Parasitology, Sichuan Agricultural University (Sichuan, China). About 300 mites, consisting of pooled larvae, nymphs, and adults, were collected and processed for total RNA extraction.

### Expression and purification of two recombinant serpin proteins

Total RNA was converted into cDNA using the PrimeScript RT reagent kit with gDNA Eraser (TaKaRa, Dalian, China). The two serpin genes were amplified from cDNA using the following primers: 5′-CGG GAT CCG CTC ATG TTG GTC AAC ATC-3′ (forward) and 5′-CCA AGC TTT TAA AAA TCA TGA ATT TCA CC-3′ (reverse) for Pso c 27, where the underlining indicates restriction enzymes *Bam*HI and *Hind*III; and 5′-CGG GAT CCT GAA TGC GAA TTC ATT GCT G-3′ (forward) and 5′-CCC TCG AGT CAA AAT CCA TGC ATT TCA CC-3′ (reverse) for PsoSP2, where the underlining indicates the restriction enzymes *Bam*HI and *Xho*I. The cDNA fragments were sub-cloned into the pET32a(+) plasmid (Invitrogen, Beijing, China). The recombinant proteins were expressed in *Escherichia coli* BL21 (DE3) and purified as described previously by Gu et al. [2]. The eluted fractions were concentrated using an Amicon Ultra Centrifugal Filter unit (Millipore, Billerica, MA, USA). Two purified serpin proteins were detected by 12% sodium dodecyl sulfate-polyacrylamide gel electrophoresis (SDS-PAGE).

### Sequence analysis

The DNAMAN software package version 7.0 was applied to compare the similarity between paralogous genes, and the SignalP 5.0 tool (http://www.cbs.dtu.dk/Services/SignalP/) was used to predict signal peptides. Transmembrane regions were analyzed using the Transmembrane Prediction Server (http://www.cbs.dtu.dk/services/TMHMM-2.0). B-cell epitopes were predicted by BaCelLo (http://gpcr.biocomp.unibo.it/bacello/pred.htm). The serine protease inhibition domains were analyzed using Inter-ProScan EMBL-EBI software (http://pfam.xfam.org/). Amino acid sequences were aligned using MEGA5 [13]. Secondary structure predictions were performed by JPred 4.0 (http://www.compbio.dundee.ac.uk/jpred/). The neighbor-joining (NJ) tree, including values of 1000 replications resampled tests, was constructed by MEGA5 software [13].

### Rabbit sera

Fifty *P. ovis* var. *cuniculi*-positive rabbit sera were collected from a farm located in Chengdu, Sichuan, China. Positivity for *P. ovis* var. *cuniculi* was confirmed in all rabbits based on the observation of ear scabs and micropscopic study of skin scrapings [14]. Twenty-five negative sera from *P. ovis* var. *cuniculi*-free rabbits were obtained from a farm without a history of psoroptic mange. For cross-reaction testing, an additional 30 sera samples, including *Sarcoptes scabiei*-positive sera, *Eimeria* spp.-positive sera, and *Cysticercus pisiformis*-positive sera (ten samples of each) were provided by the Department of Parasitology, Sichuan Agricultural University.

### Preparation of polyclonal antibodies and western blotting

Polyclonal antibodies were obtained by experimental immunization with purified rPso c 27 and rPsoSP2, respectively. The products were raised following the procedures of Gu et al. [2], with slight modification. Briefly, rabbits were immunized with about 1 mg purified recombinant protein four times by subcutaneous injection. Sera were collected from the marginal ear vein before immunization and 7 days after the fourth infection, and then purified by HiTrap Protein A affinity chromatography (Bio-scale™ Mini UNOsphere SUPrA^TM^ cartridge; Bio-Rad, Hercules CA, USA) to obtain the immunoglobulin G (IgG) of anti-rPso c 27 and anti-rPsoSP2.

Two purified recombinant proteins were separated by 12% SDS-PAGE and transferred to the nitrocellulose membranes using Trans-Blot SD Semi-Dry Transfer Cell (Bio-Rad). The membranes were then blocked using 5% skimmed milk powder for 2 h, following which they were washed three times (each wash 5 min) in TBST (0.02 M Tris-HCl, pH 7.6, 0.15 M NaCl, 0.05% Tween-20) and then incubated with rabbit anti-*P. ovis* var. *cuniculi* antibody, anti-rPso c 27 IgG, or anti-rPsoSP2 IgG (1:150 v/v) overnight at 4 °C. Non-infested rabbit serum was used as a negative control. After washing three times with TBST, membranes were incubated with horseradish peroxidase (HRP)-conjugated goat anti-rabbit antibody (1:1000 dilution; Boster Bio-Project Co. Dalian, China) for 1 h at room temperature. Following another three washes with TBST, the signal was detected using an Enhanced HRP-DAB Chromogenic Substrate Kit (Tiangen, Beijing, China).

### Immunolocalization of two serpin proteins in adult female *P. ovis* var. *cuniculi*

The immunolocalization of two serpin proteins were performed as previously described [15]. Briefly, adult female mites were collected, embedded in paraffin blocks, and sliced into 5-μm histological sections. The sections were then treated with 0.01 M citrate buffer and incubated with purified rabbit anti-rPso c 27 IgG, anti-rPsoSP2 IgG, or pre-immune IgG (1:200 v/v). After three washes with phosphate buffered saline (PBS), sections were incubated with fluorescein isothiocyanate-conjugated goat anti-rabbit IgG (1:200 v/v) and visualized using a fluorescent microscope (BX53; Olympus Corp., Tokyo, Japan).

### Transcriptional profiles of serpin at different life stages of *P. ovis* var. *cuniculi*

Total RNA was extracted from larvae, nymph, and adult (male/female) mites, respectively, using a MiniBest Universal RNA Extraction kit (Takara Bio Inc., Shiga, Japan). Relative gene expression was evaluated by a two-step quantitative real-time PCR (qRT-PCR) using primers (i) Pso c 27: 5′-TGG CAG CAG TGG ATC AGA ATC ATC-3′ (forward) and 5′-AAT GCA ACA GCA ACA CTG TAT GGC-3′ (reverse); PsoSP2: 5′-TCC TAC ATA CAC GTC CAT CAA CA-3′ (forward) and 5′-TGG TAC AAT AGC GAC GGC TG-3′ (reverse). The β-actin gene was used as a housekeeping control to correct for the relative fluorescence signal of the target genes, using primers 5′-TGA ATT GCC TGA TGG TCA AG-3′ (forward) and 5′-TGG CGA ACA AGT CTT TAC GG-3′ (reverse). Gene transcription was assessed according to the manufacturers’ recommendations of the real-time PCR system (LightCycler® 96 System; Roche Group, Basel, Switzerland) and the SYBR Premix Ex Taq II kit (TaKaRa). Each sample was performed in triplicate. An equal volume of ddH_2_O replaced the template cDNA as a blank control. Thermal cycling was performed as follows: one cycle at 95 °C for 30 s, 95 °C for 5 s, 58 °C for 30 s; followed by 40 cycles at 95 °C for 5 s, 59 °C for 15 s, and 95 °C for 1 s. Melting curves were plotted, and relative expression levels of the target genes were calculated by the 2^−ΔΔCt^ method.

### Establishment of an indirect ELISA (iELISA)

The establishment of iELISA was performed as described by Crowther [16]. The concentrations of antigen and primary serum samples were determined by the checkerboard titration tests. Briefly, the purified proteins were diluted twofold in 0.1 M carbonate buffer (pH 9.6) to the different concentrations and placed in 96-well plates at 100 μl/well overnight at 4 °C. The plates were washed three times in PBS containing Tween-20 (PBST; pH 7.4) (5 min per wash), then incubated first with 5% (w/v) skimmed milk powder at 37 °C for 90 min, then with 100 μl of the two-fold gradient dilution of *P. ovis* var. *cuniculi*-positive and -negative serum samples (dilution ranging from 1:20 to 1:320) at 37 °C for 1 h. The plates were subsequently washed 3 times and incubated 1 h at 37 °C with 100 μl HRP-labeled goat anti-rabbit IgG (1:3000 dilution with 0.01 M PBS) (Boster Bio-Project Co.). After 4 washes, 100 μl of TMB chromogenic solution (Tiangen, Beijing, China) was added at 37 °C for 20 min, then the reaction was stopped with 100 μl/well of 2 M H_2_SO_4_. Optical densities (OD) were read at 450 nm by a microplate reader (Thermo Fisher Scientific, Waltham, MA, USA). The optimal working conditions were determined based on the highest positive : negative serum value. The cut-off value of iELISA was determined as the mean OD_450_ value plus three standard deviations (SD) using 25 negative serum samples from naïve rabbits [2].

To further evaluate the feasibility of the iELISA, 50 *P. ovis* var. *cuniculi*-positive serum samples were assessed by the iELISA, and the sensitivity was calculated as (ELISA-positive samples × 100)/true *P. ovis* var. *cuniculi*-positive serum samples [2]. Thirty serum samples from rabbits infected with *S. scabiei*,* Eimeria* spp., and *C. pisiformis*, respectively (10 samples for each species) were used to evaluate the cross-activity of the iELISA. Twenty-five *P. ovis* var. *cuniculi*-negative serum samples from naïve rabbits and 30 serum samples in the cross-activity assay were used to determine the specificity of the iELISA, which was calculated as (ELISA-negative samples × 100)/true *P. ovis* var. *cuniculi*-negative serum samples [2]. The area under the receiver operating characteristic curve (AUC), a graph of the sensitivity (true positive rate) *versus* 1-specificity (false positive rate), was the calculated using MedCalc 19.0.7 [17].

The repeatability (intra-assay variability) and reproducibility (inter-assay variability) of the iELISA were evaluated using three *P. ovis* var. *cuniculi*-positive serum samples, substantially as previously described [18].

### The experimental infestation of rabbits with *P. ovis* var. *cuniculi* and serological testing using the newly developed iELISA

Rabbits infected with *P. ovis* var. *cuniculi* were treated strictly as previously described [2]. Briefly, ten 3-month-old naive New Zealand rabbits (5 females, 5 males) were infested with *P. ovis* var. *cuniculi*, and three non-infested rabbits were used as controls. Serum samples from 13 rabbits were collected at weeks 0, 1, 2, 3, and 4. Afterwards, a total of 65 serum samples (50 from the *P. ovis* var. *cuniculi*-infestated rabbits and 15 from the non-infested rabbits) were examined by the newly developed optimal iELISA method. Each serum sample was tested in triplicate and analyzed in one ELISA plate, with positive and negative controls also contained in the plate.

### Statistical analysis

All data are presented as the mean ± SD. And statistical differences between groups were evaluated using Mann-Whitney U-tests in SPSS software v.17.0. (IBM Corp. Armonk, NY, USA). *P* values of < 0.05 were considered to be statistically significant.

## Results

### Sequence analyses of the two serpins

The 1572-bp open reading frame (ORF) in Pso c 27 cDNA (GenBank: MT707535) encodes 523 amino acids (aa), and the 723-bp ORF in PsoSP2 cDNA (GenBank: MT707536) encodes 240 aa. The Pso c 27 protein contains a signal peptide but no transmembrane region, whereas PsoSP2 appears to contain a transmembrane region but no signal peptide.

**Fig. 1:**
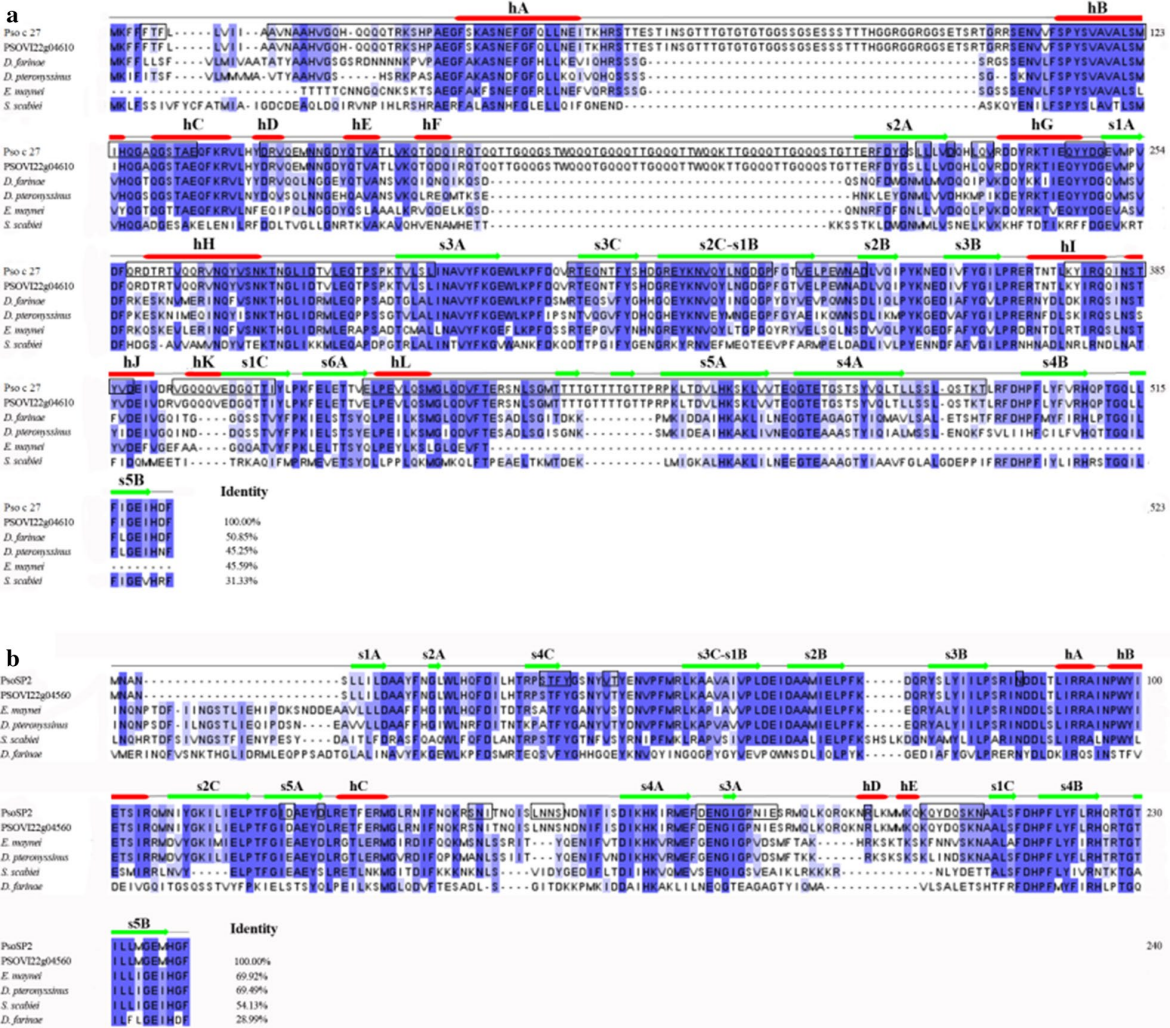
Multiple sequence alignment of *Psoroptes ovis* var. *cuniculi* serpin genes Pso c 27 (**a**) and PsoSP2 (**b**). **a** Pso c 27. Multiple sequence alignment of the deduced amino acid sequence of Pso c 27 with homologous sequences of related proteins of other parasites: *P. ovis* (PSOVI22g04610), *Dermatophagoides farinae* (GenBank: AIO08851.1), *D. pteronyssinus* (GenBank: ATI08940.1), *Euroglyphus maynei* (GenBank: OTF72764.1), and *Sarcoptes scabiei* (GenBank: AEB40052.1). **b** PsoSP2. Multiple sequence alignment of the deduced amino acid sequence of PsoSP2 with homologous sequences of related proteins of other parasites: *P. ovis* (PSOVI22g04560), *Euroglyphus maynei* (GenBank: OTF74296.1), *Dermatophagoides pteronyssinu*s (GenBank: XP_027200949.1), *Sarcoptes scabiei* (GenBank: KPM10873.1), and *Dermatophagoides farinae* (GenBank: AAP35082.1). *Psoroptes ovis* (PSOVI22g04610) and *P. ovis* (PSOVI22g04560) were obtained from the Online Resource for Community Annotation of Eukaryotes (OrcAE) (https://bioinformatics.psb.ugent.be/orcae/overview/Psovi). Helices are indicated with red horizontal bars. Horizonatal dark-green arrows along the sequence indicate sheets. Elements of secondary structure are labeled as:* hA*,* hB*, etc. A-helix, B-helix, etc.,* s1A*,* s2A*, etc. strand 1 of the A β-sheet, strand 2 of the A β-sheet, etc. Consistent residues are highlighted with a dark-blue background, and consistent partial residues are highlighted with a light-blue background. B-cell epitopes are marked with a black box

**Fig. 2:**
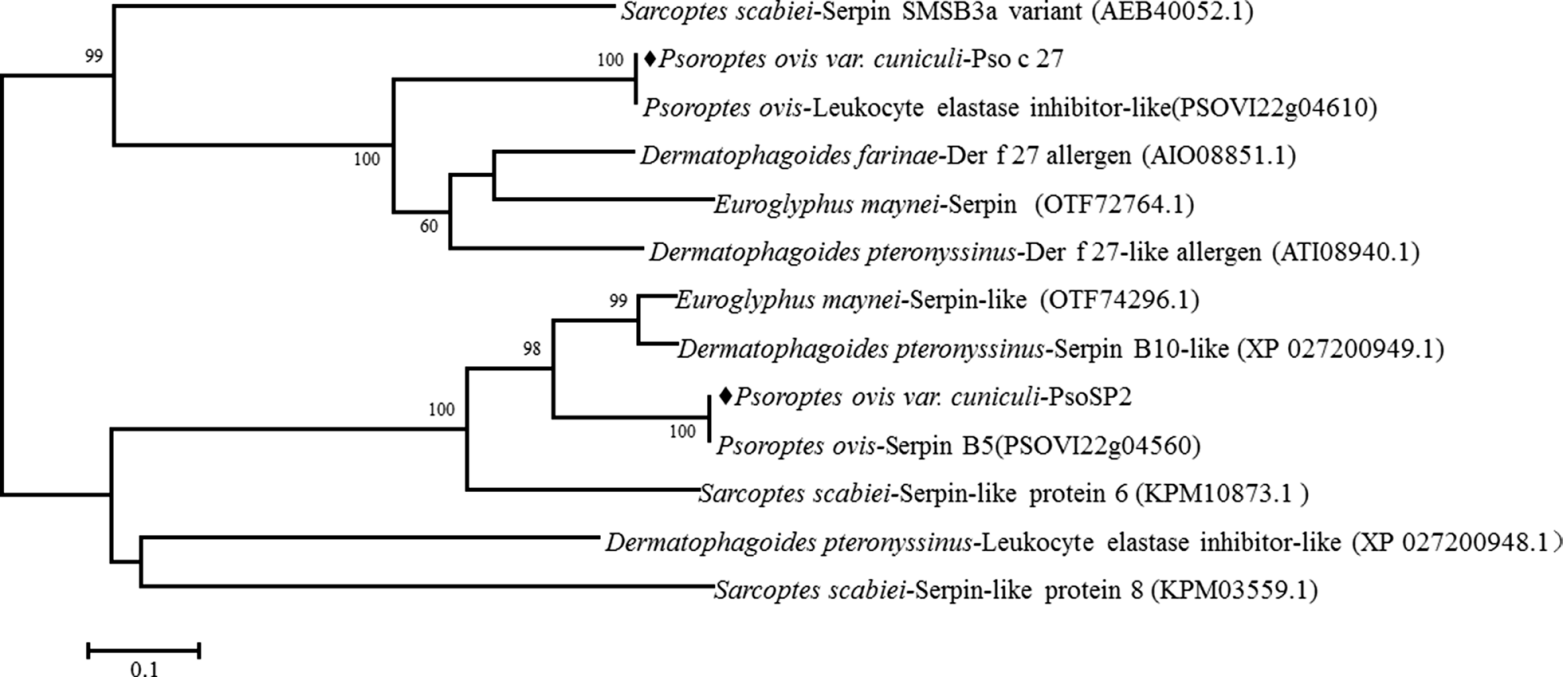
The neighbor-joining tree was constructed based on the deduced amino acid sequence of serpin. The numbers at nodes are the bootstrapping frequency (Bf) values of 1000 replications

Pso c 27 and PsoSP2 shared 31.33–50.85 and 28.99–69.92% aa sequence identity with orthologs in other mites (Fig. 1). Interestingly, Pso c 27 and PsoSP2 shared 100% aa sequence identity with the reported serpin-like proteins of *P. ovis* PSOVI22g04610 and PSOVI22g04560, respectively [19] (Fig. 1). Pso c 27 consisted of 12 helices and three sheets, while PsoSP2 consisted of five helices and three sheets (Fig. 1a). A serpin domain was identified in the aa sequence from Arg78 to His493 for Pso c 27 and from Asn4 to Met237 for PsoSP2 [20]. Moreover, both proteins appeared to possess the specific serpin signature at the deduced aa sequences from 496 to 506 (LRFDHPFLYFV) for Pso c 27 and from 213 to 223 (LSFDHPFLYFL) for PsoSP2, respectively (Fig. 1a, b). The NJ tree revealed that Pso c 27 had the closest relationship with *P. ovis-*leukocyte elastase inhibitor-like protein (PSOVI22g04610), then clustered with *D. farinae*-Der f 27 allergen, *D. pteroyssinus*-Der f 27-like allergen, and *E. maynei*-serpin (bootstrap frequency [Bf] = 100%), whereas PsoSP2 had the closest relationship with *P. ovis* serpin B5 (PSOVI22g04560) (Bf = 100%), then grouped with *E. maynei*-serpin-like and *D. pteroyssinus*-serpin B10-like (Bf = 98%; Fig. 2).

### Expression and identification of two recombinant serpins

**Fig. 3:**
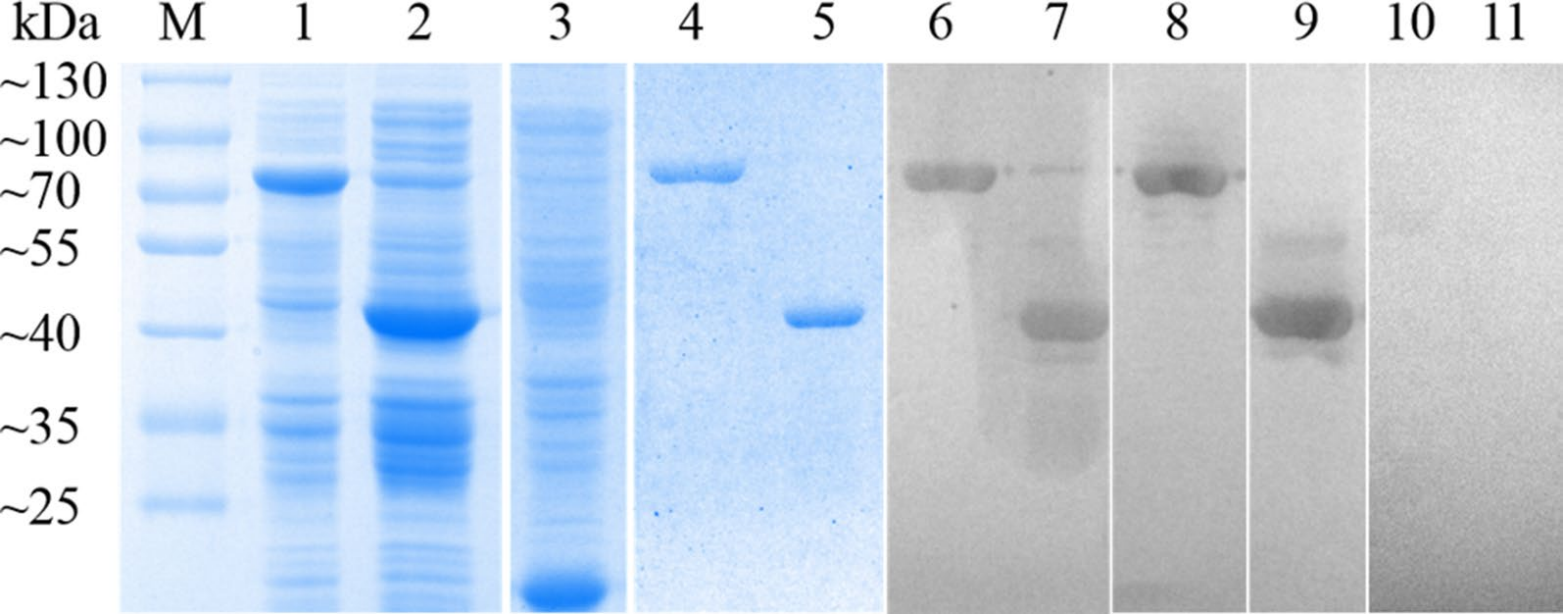
Immunoblotting with the serum-binding recombinant Pso c 27 and PsoSP2.* Lanes M* Protein molecular weight marker,* 1* recombinant proteins of *Escherichia coli* expressing pET32a(+)-Pso c 27 induced by the reagent IPTG,* 2* recombinant proteins of *E. coli* expressing pET32a(+)-PsoSP2 produced by IPTG,* 3* proteins of *E. coli* expressing pET32a(+),* 4* purified rPso c 27,* 5* purified rPsoSP2,* 6* purified rPso c 27 immunoblotted with *P. ovis* var. *cuniculi*-positive serum from rabbits with psoroptic mange,* 7* purified rPsoSP2 immunoblotted with *P. ovis* var. *cuniculi*-positive serum from rabbits with psoroptic mange,* 8* purified rPso c 27 immunoblotted with anti-rPso c 27 immunoglobulin (IgG),* 9* purified rPsoSP2 immunoblotted with anti-rPsoSP2 IgG,* 10* purified rPso c 27 immunoblotted with *P. ovis* var. *cuniculi*-negative serum,* 11* purified rPsoSP2 immunoblotted with *P. ovis* var. *cuniculi*-negative serum

rPso c 27 were mainly present in the supernatant, with an expected size of ~ 75 kDa, whereas rPsoSP2 principally present in insoluble inclusion bodies, with an expected size of ~ 46 kDa (including ~ 18 kDa His-tag fusion peptide from pET-32a) (Fig. 3). Western blotting showed that rPso c 27 and rPsoSP2 reacted with both *P. ovis* var. *cuniculi*-positive sera and the correspondent anti-serum IgG from rabbits, but not with negative sera, revealing a favourable reactivity and antigenicity (Fig. 3).

### Tissue localization of two serpins in adult female *P. ovis* var. *cuniculi*

**Fig. 4:**
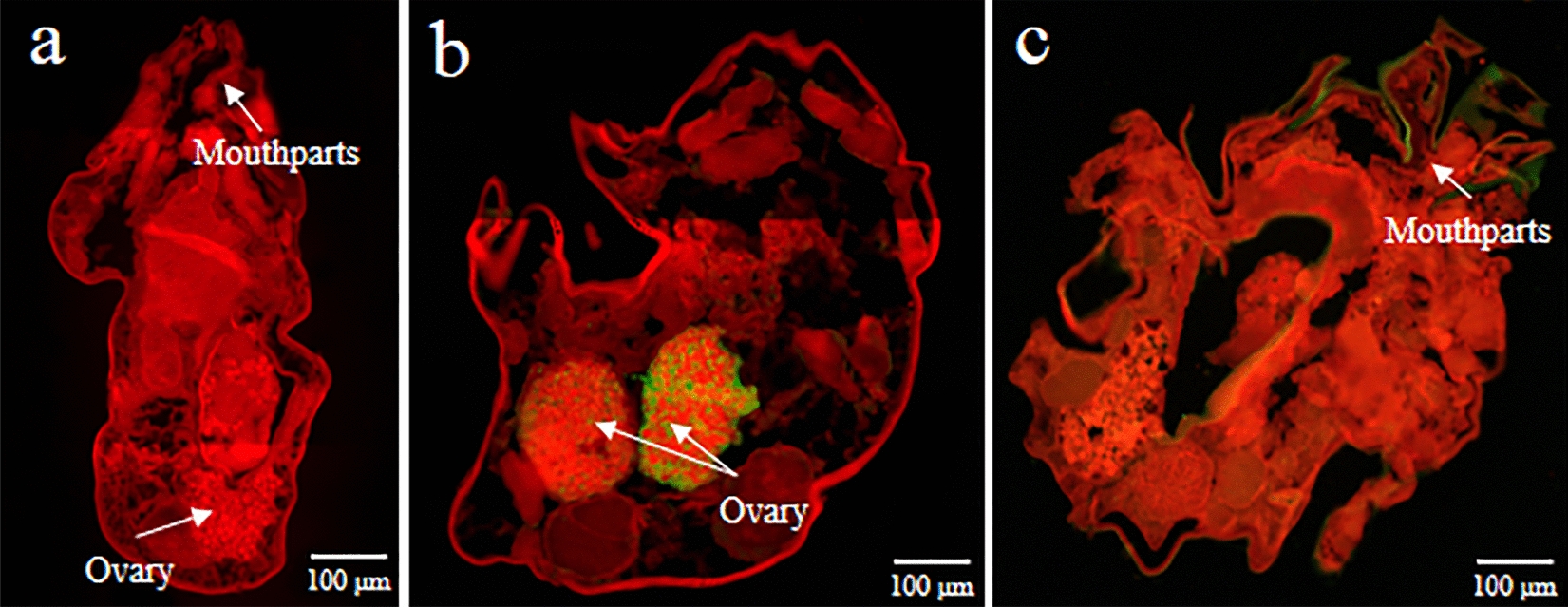
Immunolocalization of Pso c 27 and PsoSP2 in the adult female of *P. ovis* var. *cuniculi*. **a** Incubation with the negative IgG of the rabbit before immunization, **b** incubation with the specific IgG of anti-rPso c 27, **c** incubated with the specific IgG of anti-rPsoSP2. All images were taken under a fluorescent microscope (magnification 100×)

Native Pso c 27 and PsoSP2 were located in the ovary and mouthparts of female mites, respectively (Fig. 4b, c). No fluorescence signal was observed in adult female mites using pre-immunized rabbit IgG antibodies (Fig. 4a).

### Transcriptional profiles of the two serpins in *P. ovis* var. *cuniculi*

**Fig. 5:**
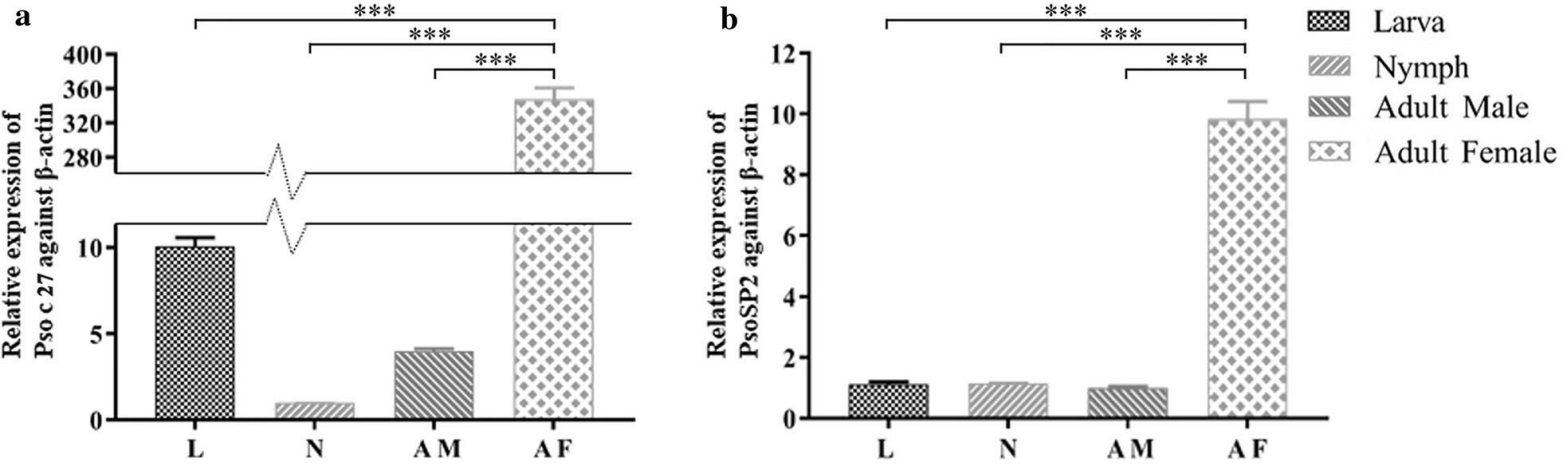
Relative transcriptional profiles of Pso c 27 (**a**) and PsoSP2 (**b**). The internal reference gene was β-actin. Data are represented as the mean with standard deviation (SD) from triplicate measures.* L* Larva,* N* nymph,* AM* adult male,* AF* adult female. Asterisks (***) indicate a statistically significant difference at *P* < 0.0001

The qRT-PCR data revealed that Pso c 27 and PsoSP2 cDNAs were expressed throughout the life-cycle of mites but that there was significantly higher expression in female mites than in larvae, nymphs, and male mites, respectively (Pso c 27, *F*_(3, 8)_ = 1935.953, *P* < 0.0001; PsoSP2, *F*_(3, 8)_ = 660.669, *P* < 0.0001) (Fig. 5).

### Serodiagnosis potential of two recombinant serpin proteins

Using checkerboard titration, we determined that the optimal working conditions of the iELISA were 46.0 μg/ml of rPso c 27, 64.5 μg/ml of rPsoSP2 for coated antigens, and a 1:100 dilution for rabbit sera. The cut-off values of OD_450_ were 0.633 for rPso c 27 and 0.490 for rPsoSP2.

**Fig. 6:**
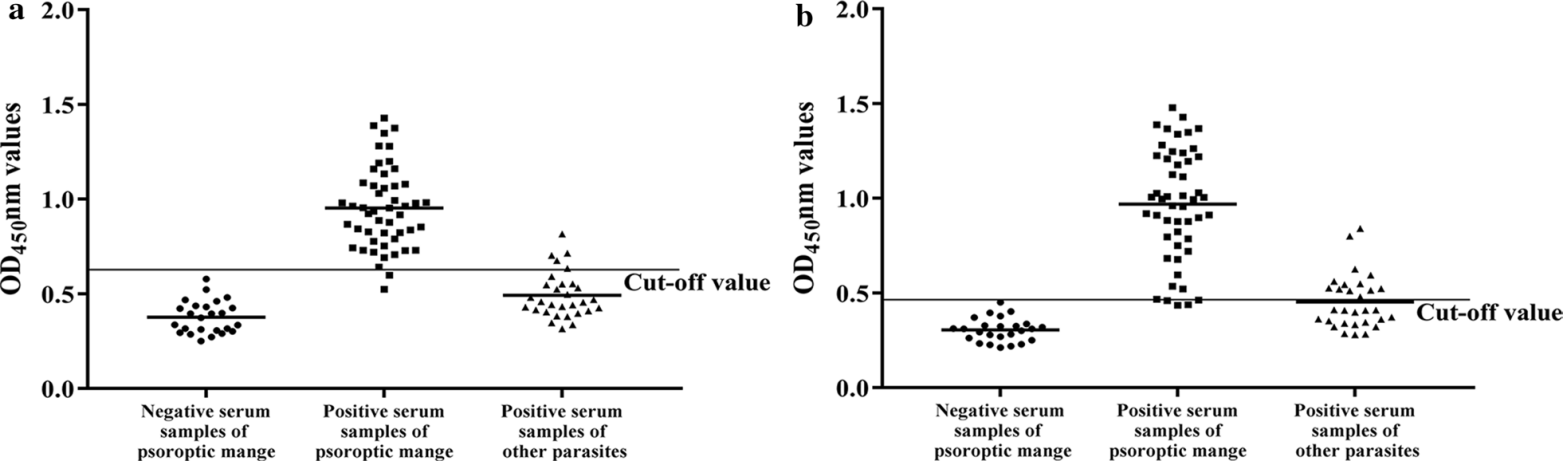
Specificity, sensitivity,and cross-reactivity of rPso c 27 (**a**) and rPsoSP2 (**b**) by the newly developed indirect enzyme-linked immunosorbent assay (iELISA). The thin horizontal line represents the cut-off value (rPso c 27-iEISLA: 0.633; rPsoSP2-iELISA: 0.490). Statistically significant differences were compared between *P. ovis* var. *cuniculi*-positive serum and the other serum samples, including *Eimeria* spp.-positive, *C. pisiformis*-positive, *S. scabiei*-positive and *P. ovis* var. *cuniculi-*negative serum samples.* OD* Optical density. Asterisks (***) indicate a statistically significant difference at *P* < 0.0001

**Fig. 7:**
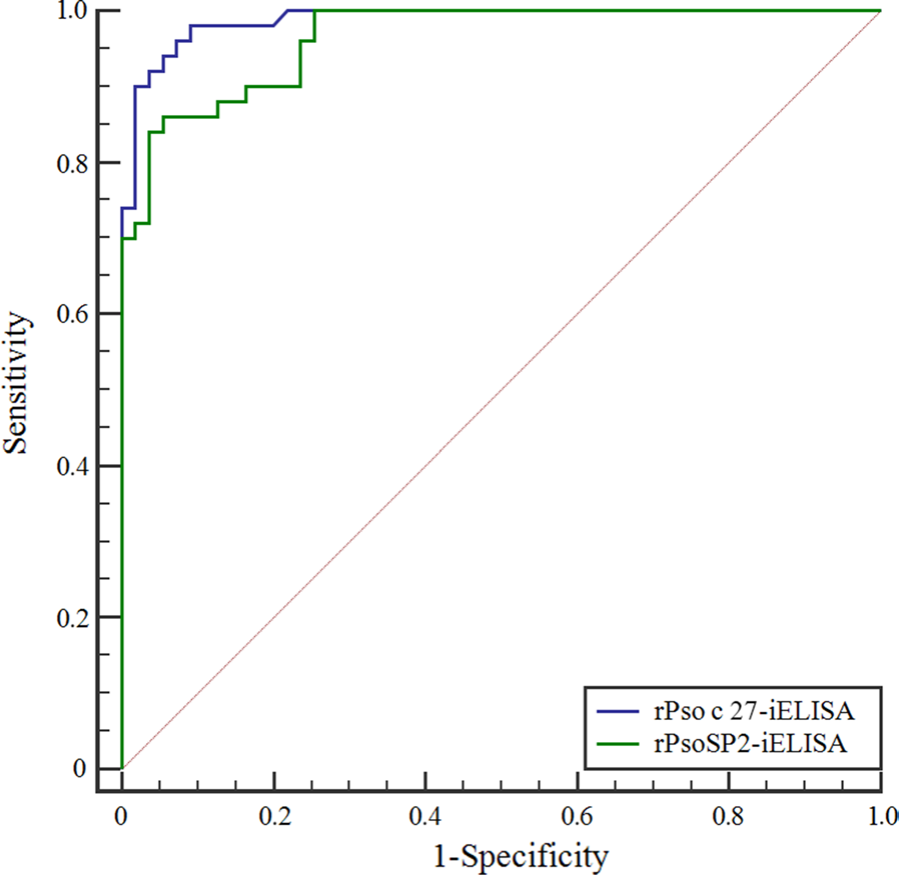
The receiver operating characteristic (ROC) curves of the rPso c 27-iELISA and rPsoSP2-iELISA for the detection of antibodies against *P. ovis* var. *cuniculi*. The ordinate represents the sensitivity of the iELISA. The abscissa represents the 1-specificity of the iELISA. The purple line shows the mean area under the curve (AUC) plot of rPso c 27-iELISA, and the green line shows the mean AUC plot of rPsoSP2-iELISA

The sensitivities were determined as the results of *P. ovis* var. *cuniculi*-positive sera, with 96.0% sensitivity for rPso c 27 (48/50) (Fig. 6a) and 90.0% for rPsoSP2 (45/50) (Fig. 6b). The specificities were 90.91% for rPso c 27 (50/55) and 78.18% for rPsoSP2 (43/55). Consequently, the AUC was 0.988 for rPso c 27-iELISA (95% confidence interval [CI] CI 0.944–0.999) and 0.964 for rPsoSP2-iELISA (95% CI 0.908–0.991), indicating that the rPso c 27-iELISA showed a better accuracy to detect specific antibodies against *P. ovis* var. *cuniculi* than the rPsoSP2-iELISA (Fig. 7). The intra- and inter-assay variabilities of rPso c 27-iELISA were < 5%, indicating that the newly developed rPso c 27-iELISA was stable and reproducible.

### Serodiagnostic test of rabbits experimentally infested with *P. ovis* var. *cuniculi*

**Fig. 8:**
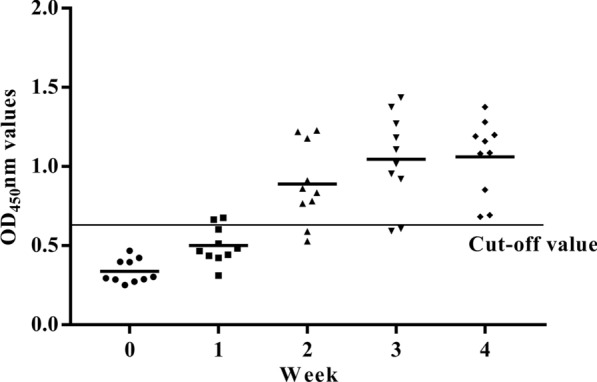
Serum antibody profiles detected by rPso c 27-iELISA in rabbits experimentally infected with *P. ovis* var. *cuniculi*. The ordinate represents the OD_450_ value of serum. The abscissa represents the serum of different infection period. The thin horizontal line represents the cut-off value (rPso c 27-iELISA: 0.633)

At 4 weeks post-infestation (p.i.), all infested rabbits were observed to have ear scabs. Skin scrapings also tested positive for *P. ovis* var. *cuniculi*. By rPso c 27-iELISA, the mean value of the anti-rPso c 27 level from the infestation group revealed an increase from 1 to 4 weeks p.i. (Fig. 8). In the infestion group, anti-rPso c 27 positivity above the cut-off value was first detected in two of ten serum samples at 1 week p.i., following which the positivity gradually increased to 80% of samples (8/10) at 2 and 3 weeks p.i., then up to 100% of samples (10/10) at 4 weeks p.i. (Fig. 8). In the non-infestation group, the rate of anti-rPso c 27 antibody positivity remained below the cut-off value throughout the experiments.

## Discussion

In the present study, two *P. ovis* var. *cuniculi* serpins were characterized, and the potential of their recombinant proteins was evaluated for serodiagnosis of *P. ovis* var. *cuniculi* infestation in rabbits. The *Psoroptes* mites used in the present study and our previous transcriptomic study are the same species, and the present findings confirmed the transcriptomic results of the earlier study in showing that two cloned serpin ORFs had 100% identity with the assembled serpin sequences in the transcriptome data [12]. The predicted aa sequence showed the low overall identity of serpins compared those in other mites; however, these two target proteins were identified as typical serpins due to the presence of such features as the serpin domain and serpin signature in the C-terminal end [20]. Pso c 27 shared 50.85% aa sequence identity with the newly characterized *D. farinae* Der f 27 allergen, which has been proven to orchestrate the pulmonary inflammatory response and mediate a Th2-type response in mice [21]. In addition, NJ analysis revealed that Pso c 27 is closely related to Der f 27. Based on the homology results and the genetic relationship between Pso c 27 and Der f 27, Pso c 27 may be considered to be an allergen of *P. ovis* var. *cuniculi* that is possibly associated with the instigation of the host cutaneous pro-inflammatory response [22]. This cutaneous inflammation has been shown to result in serum extravasation, thereby providing sufficient food for mite population growth, and to aggravate scabby lesions [5, 14]. The expression of Pso c 27 and PsoSP2 in all stages of mites indicated that Pso c 27 and PsoSP2 possibly play an essential role in the development of *P. ovis* var. *cuniculi*. However, significant differences were seen for the transcription of Pso c 27 in the different life-cycle stages, and female mites showed the highest level of expression, with 347-fold change. In addition, the native protein was located in the ovary of female mites, indicating that Pso c 27 is possibly an essential factor in vitellogenesis [19, 23]. This role of serpin in vitellogenesis has been proven in a recent study, which indicated that RNA interference (RNAi) of the serpin gene resulted in a reduction of yolk granule accumulation in *Rhipicephalus haemaphysaloides* [24].

*Psoroptes* mites are serum-feeding ectoparasites [5] and possess the ability to counter the host’s complement attack. In this study, PsoSP2 showed homology to the *S. scabiei* serpin family genes (20.98–54.13% aa sequence identity), some of which have been confirmed to inhibit the activation of complement pathways [25, 26]. Moreover, the native PsoSP2 localized in the mouthparts of female mites and its cDNA expression throughout the life stages of mites suggest that PsoSP2 may appear to be vital in mites for anti-complement activity to successful serum-feeding [5, 9], and PsoSP2 could be a potential vaccine candidate.

Psoroptic mange spreads rapidly under crowded conditions and causes major morbidity in commercial rabbit husbandry in China [27]. Thus, timely diagnosis and treatment of *P. ovis* var. *cuniculi* infestation in rabbits are of paramount importance to reduce the risk of disease transmission and improve profitability. In China, the current diagnostic method for this disease, by examination of skin scrapings under the microscope, is extremely time-consuming and inefficient, especially for low densities of mites and sub-clinical infestations in rabbits. Thus, it is imperative to develop effective immunoreactive antigens for the rapid and accurate diagnosis of *P. ovis* var. *cuniculi* infestation in rabbits. Rabbits infested with *P. ovis* var. *cuniculi* can evoke a sero-specific antibody [1, 28], and this sero-specific antibody has been induced at the early phase of parasite infestation when rabbits appeared asymptomatic [1, 2]. Thus, the ELISA can be considered as an accurate method for detecting carriers of low densities of mites and/or sub-clinical infestations when compared with the microscopy of skin scrapings. In a previous study, serpin of *Schistosoma mansoni* was considered to be a promising species-specific diagnostic antigen in human schistosomiasis [10]. Therefore, in this study, we evaluated the serodiagnostic potential of rPso c 27 and rPsoSP2 by developing an iELISA. Compared to the rPsoSP2-iELISA, the rPso c 27-iELISA displayed better diagnostic efficiency, with higher values for sensitivity, specificity, and AUC (rPso c 27-rPsoSP2: 96.0–90.0, 90.91–78.18, and 0.988–0.964, respectively). Although rPso c 27 did show cross-reaction with sera from three of ten serum samples with *S. scabiei *infestations, the cross-reaction between these two ectoparasites has been commonly shown in other studies [2, 29]. Fortunately, these two mite species were effectively treated with the same acaricide [3, 30]. Also, one in ten rabbits infested with *S. scabiei*, *C. pisiformis*, or *Eimeria* spp. showed a sero-reaction with rPso c 27; however, their OD values were close to the cut-off value and appeared to markedly lower than those rabbits infested with *P. ovis* var. *cuniculi* (*F*_(1, 78)_ = 115.444, *P* < 0.0001). Moreover, the rPso c 27-iELISA was able to detect seropositivity in 80% (8/10) of rabbits as early as week 2 p.i., prior to visible clinical signs and microscopy-positive skin scrapings. In terms of its high sensitivity and specificity, Pso c 27 can be considered to be the more suitable candidate antigen for serodiagnosis of *P. ovis* var. *cuniculi* infestation in rabbits, especially at the early stage of infestation.

## Conclusions

In conclusion, Pso c 27 and PsoSP2 cDNAs displayed the typical characterization of the serpin superfamily, possessing the regular serpin domain and signature. The gene expression of Pso c 27 and PsoSP2 were found during all life stages of mites, with a significantly high expression in adult female mites. Compared to rPsoSP2, rPso c 27 seemed to display a better diagnostic efficiency than PsoSP2 by iELISA, suggesting that Pso c 27 could be developed as a potential antigen for serological diagnosis of *P. ovis* var. *cuniculi* infestation in rabbits, especially at the early stage of infestation.

## Data Availability

The nucleotide sequences of serpin genes from *P. ovis* var. *cuniculi* in this article are available in the GenBank databases under the accession numbers MT707535 (Pso c 27) and MT707536 (PsoSP2). The other data supporting our findings and conclusions are available in the article.

## Abbreviations

AUC: Area under the receiver operating characteristic curve
Bf: Bootstrapping frequency
(i)ELISA: (Indirect) enzyme-linked immunosorbent assay
HRP: Horseradish peroxidase
NJ: Neighbor-joining
ORF: Open reading frame
p.i.: Post-infestation
RNAi: RNA interference
ROC: Receiver operating characteristic curve
rPso c 27/rPsoSP2: Recombinant Pso c 27/recombinant PsoSP2
serpin: Serine protease inhibitor
